# Long‐Term Effects of Freeze–Thaw Events on Ecosystem Carbon Exchange

**DOI:** 10.1002/ece3.73222

**Published:** 2026-03-11

**Authors:** Qingfeng Xu, Kai Guo, Jintao Zhao, Chunjing Zhao, Yinan Wang, Fengying Jin

**Affiliations:** ^1^ Key Laboratory of Water and Soil Conservation on the Loess Plateau of MWR Yellow River Institute of Hydraulic Research Zhengzhou China

**Keywords:** ecosystem resistance, ecosystem respiration, freeze–thaw cycles, machine learning, net ecosystem exchange, soil temperature

## Abstract

Freezing–thawing cycle (FTC) event plays a significant role in the regions with relative higher altitude and latitude, which is judged based on soil temperature. It affects soil environment and ecosystem productivity by altering soil physical structure, including physicochemical properties and water phase changes. Drawing on more than a decade of continuous measurements from 24 eddy‐covariance towers, we quantified how freeze–thaw cycles affect net ecosystem exchange (NEE) and ecosystem respiration (RE) and assessed the capacity of these carbon fluxes to withstand such events. The average percentile of ecosystem NEE in the first, middle, and last three stages of freeze–thaw events is 66.57%, 61.77%, and 57.21%, respectively. Prior to the freeze–thaw event, ecosystem respiration accounted for 28.07% of the reference level; during the event, this proportion declined to 24.38%, whereas it subsequently rose to 31.75% after the freeze–thaw disturbance. Resistance denotes an ecosystem's ability to preserve its functional stability throughout a freeze–thaw cycle. It is quantified as the ratio of the change in ecosystem carbon exchange before and during the freeze–thaw event to the corresponding change in soil temperature. Mean annual temperature (MAT), duration of freeze–thaw events, and elevation emerged as the primary drivers governing the resistance of both NEE and RE. Collectively, our results underscore how freeze–thaw cycles modulate carbon dynamics and why this interaction merits heightened attention under a changing climate.

## Graphical abstract

In this work, we utilized 24 long‐term (over 10 years) observed flux tower sites to analyze the impact of freeze–thaw events on net ecosystem carbon exchange (NEE) and ecosystem respiration (RE), and explored the resistance of ecosystem carbon exchange to freeze–thaw events.

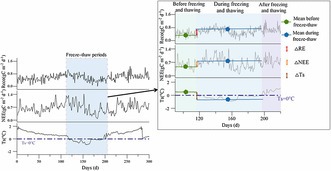

## 1 Introduction

Freeze–thaw cycle (FTC) events plays a significant role in the regions with relative higher altitude and latitude, triggered by diurnal or seasonal fluctuations in soil and atmospheric temperatures (Yu et al. 2011; Ala et al. 2016). FTC disturbances alter the soil environment through reorganization of the soil physical structure (Ma et al. 2021), encompassing microbial processes, physico‐chemical characteristics, and phase transitions of water (Ji et al. 2022; Rooney et al. 2022). Such alterations can each modulate ecosystem carbon fluxes, with a pronounced pulse of soil‐derived CO_2_ frequently documented across diverse ecosystems following a freeze–thaw cycle (Xiao et al. 2019; Gao et al. 2021; Xing et al. 2023). The permafrost zone is believed to lock away roughly 1100–1672 Pg of soil organic carbon (Hugelius et al. 2014). As the Arctic continues to warm, the winter soil freezing melting period (i.e., the frequency and intensity of start and end dates) undergoes changes (Wang et al. 2020; Rooney et al. 2022). It is expected that permafrost regions will transform into seasonal permafrost regions (Guo and Wang 2013), as permafrost melting and frequent FTC accelerate thawing of permafrost and more frequent FTC spur the breakdown of soil organic matter. Once liberated, this carbon‐rich substrate is rapidly metabolized by the resident microbial community and vented to the atmosphere as CO_2_, CH_4_, and N_2_O—powerful greenhouse gases that further intensify global warming. Consequently, scientific interest in soil freeze–thaw processes has surged (Gao et al. 2022; Yun et al. 2022; Liu et al. 2024).

Calculating the resistance of ecosystems to disturbances is a commonly used method for quantifying their response to extreme climate conditions (Li et al. 2020; Shekhar et al. 2023). Resistance (Res) is viewed as the capacity of an ecosystem to counteract performance losses inflicted by disturbances, manifesting as its ability to endure extreme events without substantial functional decline (Nimmo et al. 2015; Chang et al. 2018). Resistance indices are widely employed to quantify how ecosystems respond to climatic extremes such as droughts, heat waves, and high VPD. The concept of resistance can be adapted to different ecosystems, and its mathematical expression involves a variety of forms without a fixed form of requirement (Schwarz et al. 2020; Yao et al. 2022; Shekhar et al. 2023).

Due to objective environmental limitations, previous research on soil freeze–thaw mainly used indoor experimental methods (Song et al. 2017; Ma et al. 2021). Yet the mismatch between manipulated freeze–thaw parameters in laboratory or modeling studies (e.g., frequency, duration) and actual field conditions can markedly alter how respiration and other key processes react to FTC (Henry 2007; Wang et al. 2023). Field‐based monitoring campaigns were carried out to assess how freeze–thaw cycles affect ecosystem productivity, with a focus on the regulatory influence of soil temperature and moisture dynamics (Wang, Lv, et al. 2020; Wang et al. 2023; Xing et al. 2023). Overall, research related to the productivity of freeze–thaw on ecosystems in cold regions is still relatively scarce due to environmental conditions. Additional investigations into the interplay between freeze–thaw cycles and environmental factors are essential for deepening our insight into the chemical and biological responses of permafrost carbon cycling (Yi et al. 2015; Rooney et al. 2022).

It is imperative to sharpen our mechanistic understanding of how freeze–thaw cycles shape ecosystem productivity. It is challenging because intricate environment–climate interactions and possible long‐term ecological stress memory can jointly modulate ecosystem resistance. We use data‐driven and machine‐learning approaches to dissect how environmental or climatic drivers affect ecosystem productivity during freeze–thaw and to identify environmental or climatic interactions. Drawing on decade‐long records from 24 eddy‐covariance towers that span multiple plant functional types, this study pursues two aims: (1) it quantifies how freeze–thaw events influence global‐scale net ecosystem carbon exchange (NEE) and ecosystem respiration (RE) by characterizing both the events themselves and the accompanying local meteorological conditions and (2) to analyze the resistance of ecosystem carbon exchange to freeze–thaw events.

## 2 Data and Methods

### 2.1 Datasets

FLUXNET (http://fluxnet.fluxdata.org/) operates a worldwide eddy‐covariance network that has generated extensive datasets from over 200 monitoring sites since 1991, including water and carbon fluxes, multiple climate factors, etc., on time scales ranging from half‐hourly to yearly (Wang et al. 2022). Because freeze–thaw status must be resolved at the daily scale (Wang, Lv, et al. 2020), we screened the FLUXNET2015 daily product and retained 24 sites that each provide at least a decade of continuous observations, including precipitation (P, mm), temperature (TA, °C), incident shortwave radiation (Rg, W m^−2^), vapor pressure deficit (VPD, hPa), wind speed (WS, m^−1^), soil water content (SWC, %), soil temperature (Ts, °C), RE (μ mol CO_2_ m^−2^ s^−1^), and NEE (μ mol CO_2_ m^−2^ s^−1^). The FLUXNET dataset adopts consistent methods for quality control, data interpolation, and other techniques consistently (Chen et al. 2023). In order to avoid autocorrelation between RE and NEE caused by vortex covariance CO_2_ flux partitioning, we extracted RE and NEE from daytime partitioning outputs.

**FIGURE 1:**
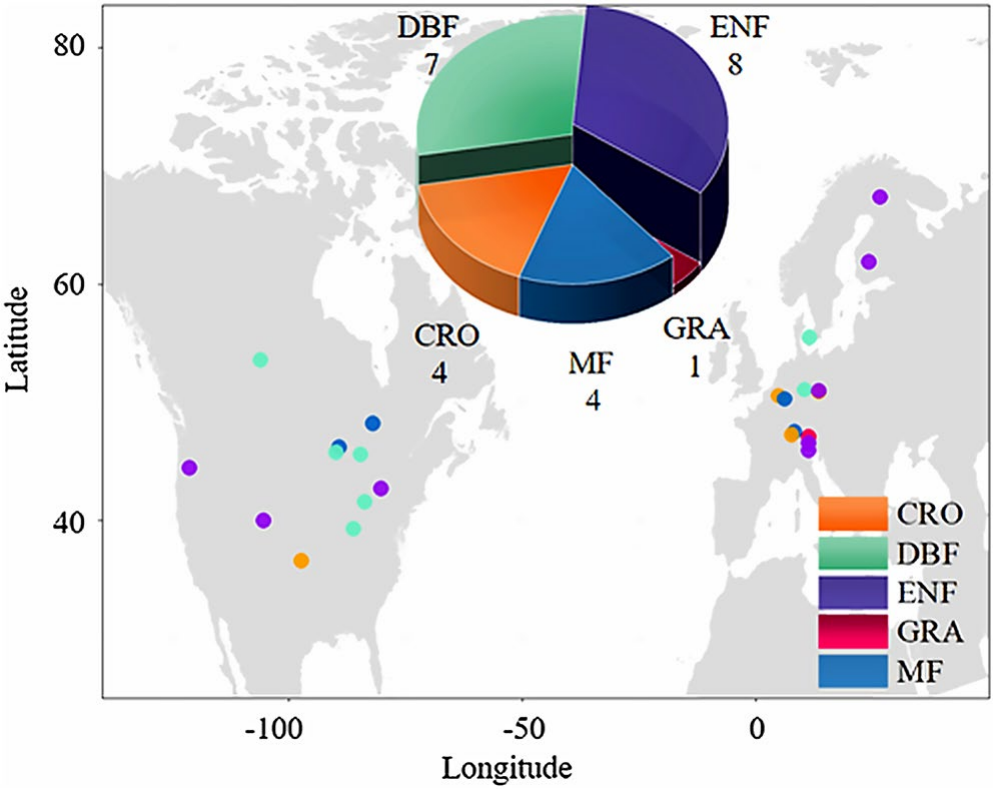
Location of 24 eddy covariance flux sites used in this study. The sites represent five different plant functional types—deciduous broadleaf forests (DBF, 7 sites), evergreen needle‐leaf forests (ENF, 8 sites), mixed forests (MF, 4 sites), grasslands (GRA, 1 sites), and croplands (CRO, 4 sites). See Table 1 for more information about each site.

This study used a total of 24 individual monitoring stations, each with a monitoring time exceeding 10 years, mainly distributed in high latitude regions of the Northern Hemisphere (Figure 1 and Table 1). Following the International Geosphere–Biosphere Programme (IGBP) scheme, the sites group into five plant functional types: deciduous broadleaf forests (DBF), evergreen needle‐leaf forests (ENF), mixed forests (MF), grasslands (GRA), and croplands (CRO).

**TABLE 1:** Site locations, vegetation classifications, and data coverage for the 24 flux towers.

Station	La (°)	Long (°)	IGBP	Elevation (m)	MAT (°C)	MAP (mm)
AT‐Neu	47.1167	11.3175	GRA	970	6.5	852
BE‐Lon	50.5516	4.7462	CRO	167	10.0	800
BE‐Vie	50.3049	5.9981	MF	493	7.8	1062
CA‐Gro	48.2167	−82.1556	MF	340	1.3	831
CA‐Oas	53.6289	−106.198	DBF	530	0.3	428.53
CA‐TP4	42.7102	−80.3574	ENF	184	8.0	1036
CH‐Lae	47.4783	8.3644	MF	689	8.3	1100
CH‐Oe2	47.2864	7.7337	CRO	452	9.8	1155
DE‐Hai	51.0792	10.4522	DBF	430	8.3	720
DE‐Kli	50.8931	13.5224	CRO	478	7.6	842
DE‐Tha	50.9626	13.5651	ENF	385	8.2	843
DK‐Sor	55.4859	11.6446	DBF	40	8.2	660
FI‐Hyy	61.8474	24.2948	ENF	181	3.8	709
FI‐Sod	67.3624	26.6386	ENF	180	−1.0	500
IT‐Lav	45.9562	11.2813	ENF	1353	7.8	1291
IT‐Ren	46.5869	11.4337	ENF	1730	4.7	809.3
US‐ARM	36.6058	−97.4888	CRO	314	14.76	843
US‐Me2	44.4526	−121.5589	ENF	1253	6.28	523
US‐MMS	39.3232	−86.4131	DBF	275	10.85	1032
US‐NR1	40.0329	−105.546	ENF	3050	1.5	800
US‐Oho	41.5545	−83.8438	DBF	230	10.1	849
US‐Syv	46.242	−89.3477	MF	540	3.81	826
US‐UMB	45.5598	−84.7138	DBF	234	5.83	803
US‐WCr	45.8059	−90.0799	DBF	520	4.02	787

Abbreviations: CRO, cropland; DBF, deciduous broadleaf forest; ENF, evergreen needleleaf forest; GRA, grassland; MF, mixed forest.

### 2.2 Methods

#### 2.2.1 Definition of Freeze–Thaw Events

**FIGURE 2:**
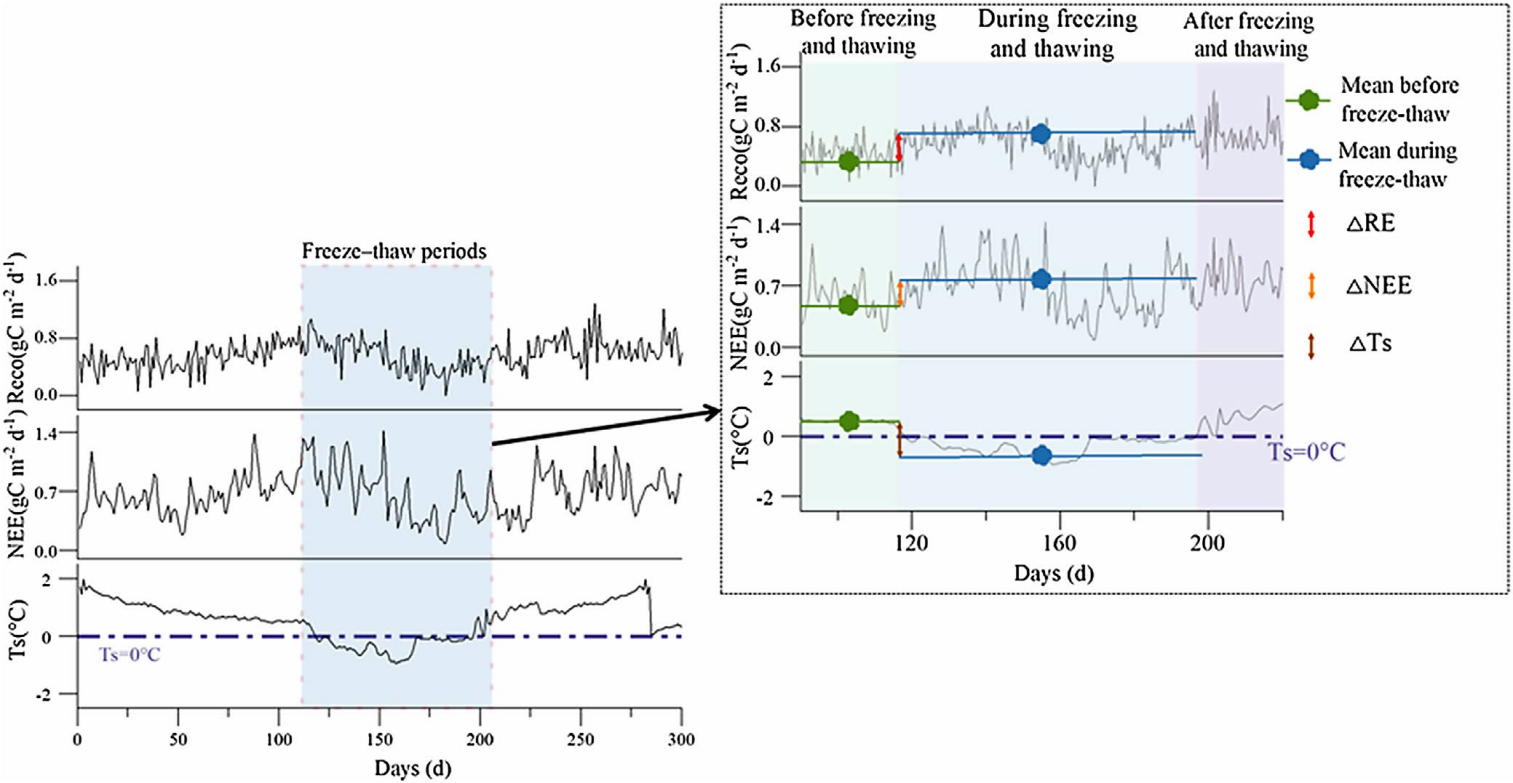
Schematic diagram of freeze–thaw event definition and associated parameter calculation. △RE, △NEE, and △Ts are defined as the difference between the values during freeze–thaw events and those before the events.

Due to significant missing data in the half‐hour monitoring, we chose to use daily scale data for freeze–thaw assessment. Based on the existing FTC definition with appropriate modifications (Wang, Lv, et al. 2020), we used the daily average temperature (ST_avg_) of shallow soil (approximately 5 cm) to define seasonal thawing and freezing. When ST_avg_ < 0°C for more than five consecutive days (freezing period), it was considered as soil freezing; when the soil was frozen and ST_avg_ > 0°C for at least five consecutive days (thawing period), it was considered that the soil has thawed (Figure 2).

#### 2.2.2 NEE and RE Resistance Estimates

In this study, based on the above judgment of the occurrence of freeze–thaw events, we also selected soil temperature, NEE and RE in the 10 days before freeze–thaw for calculating freeze–thaw resistance, since the freeze–thaw time was at least 10 days (5 days of freezing and 5 days of thawing, respectively).

(1) $${\mathrm{Rt}}_{\mathrm{NEE}}=\frac{\mathrm{NEE}-\text{PreNEE}}{T_{S}-\mathrm{Pre}T_{s}}$$

(2) $${\mathrm{Rt}}_{\mathrm{RE}}=\frac{\mathrm{RE}-\text{PreRE}}{T_{S}-\mathrm{Pre}T_{s}}$$

where NEE, RE, and T_s_ are the average value during the FTC; PreNEE, PreRE, and PreT_s_ are the average value before the FTC.

We applied the non‐parametric Mann–Kendall (M–K) test to assess temporal trends in freeze–thaw characteristics at each site. Because the M–K procedure made no distributional assumptions and is robust to outliers, it was well suited to hydrometeorological data. Earlier work has employed this method to detect trends in precipitation, temperature, runoff, and water quality (Dawood 2017; Zou et al. 2020). On the basis of the resulting *Z*‐scores, trends were classified as: significant decrease (*Z* < −1.96), significant increase (*Z* > 1.96), or no significant change (−1.96 ≤ *Z* ≤ 1.96) (Li et al. 2020).

#### 2.2.3 Deep Learning Model of Ecosystem NEE and RE Resistance to Freeze–Thaw

Tree‐based machine‐learning models rank among the most widely adopted nonlinear tools for predicting and attributing ecosystem dynamics (Green et al. 2022; Wang et al. 2022); on tabular datasets they typically surpass neural networks and other standard deep‐learning approaches in accuracy (Lundberg et al. 2020). Extreme gradient boosting XGBoost is an effective implementation of gradient enhanced decision trees. This supervised approach iteratively tunes its parameters against paired inputs and outputs to reach an optimal model and effectively prevents overfitting (Yang et al. 2021).

In this study, we used the XGBoost model to simulate ecosystem NEE and RE resistance to freeze–thaw under different driving factors, including freeze–thaw duration (days), elevation (m), mean annual temperature (MAT, °C), mean annual precipitation (MAP, mm), and ecosystem type. XGBoost relies on a suite of hyper‐parameters whose careful tuning is essential for curbing overfitting and model bloat, yet the sheer number of adjustable settings makes this task demanding (Parsa et al. 2020). Cross‐validation guided training and hyper‐parameter tuning, with the best settings chosen to minimize mean absolute error (MAE) and root‐mean‐square error (RMSE) in internal folds. The mesh optimized the parameters of max_depth, eta, and nrounds, and other parameters such as subsample, min_child_weight, etc., were fixed in the XGBoost model. The final optimized parameters were max_depth = 4, eta = 0.5, and nrounds = 20.

#### 2.2.4 SHAP‐Based Machine‐Learning Model Interpretation

SHAP translated the Shapley value from game theory into a model‐agnostic framework for explaining machine‐learning outputs (Parsa et al. 2020; Yuan et al. 2024). Every trait was treated as a potential contributor (Yang et al. 2021). SHAP tracked how altering a single feature shifts the model's loss—the gap between the predicted and expected resistance of NEE and RE. By averaging each predictor's marginal contribution across every possible subset of variables, SHAP yielded a distinct importance score for every feature and delivered a game‐theory‐grounded measure of local interactions (Wang et al. 2022).

In SHAP, a set *N* of *n* features was used to predict an output. The contribution *ϕᵢ* of each feature *i* to the model output was assigned according to its marginal contribution (Parsa et al. 2020). Guided by axioms that ensure a fair allocation, the Shapley values were computed as:

(3) $$\mathrm{\phi i}\left(v\right)=\sum_{S\subseteq N\setminus \left\{i\right\}}\frac{|S|!\left(n-|S|-1\right)!}{n!}\left(v\left(S\cup \left\{i\right\}\right)-v\left(S\right)\right)$$

where *S* denotes any subset of features that excludes feature *I*, and *|S|* is the number of participants in the subset *S*. *N\*{*i*} represents the set of elements that remain after element *i* is removed from the set *N*. *v*(*S*) is the total effect of the subset *S*. *v*(*S* ∪ {*i*}) is the total effect of the subset *S* plus participant *i*.

## 3 Results

### 3.1 Freeze–Thaw Detection at FLUXNET Stations

The 24 sites span North America and Europe and exhibit a range of vegetation types (Figure 1), with ENF being the type with the most freeze–thaw events.

**FIGURE 3:**
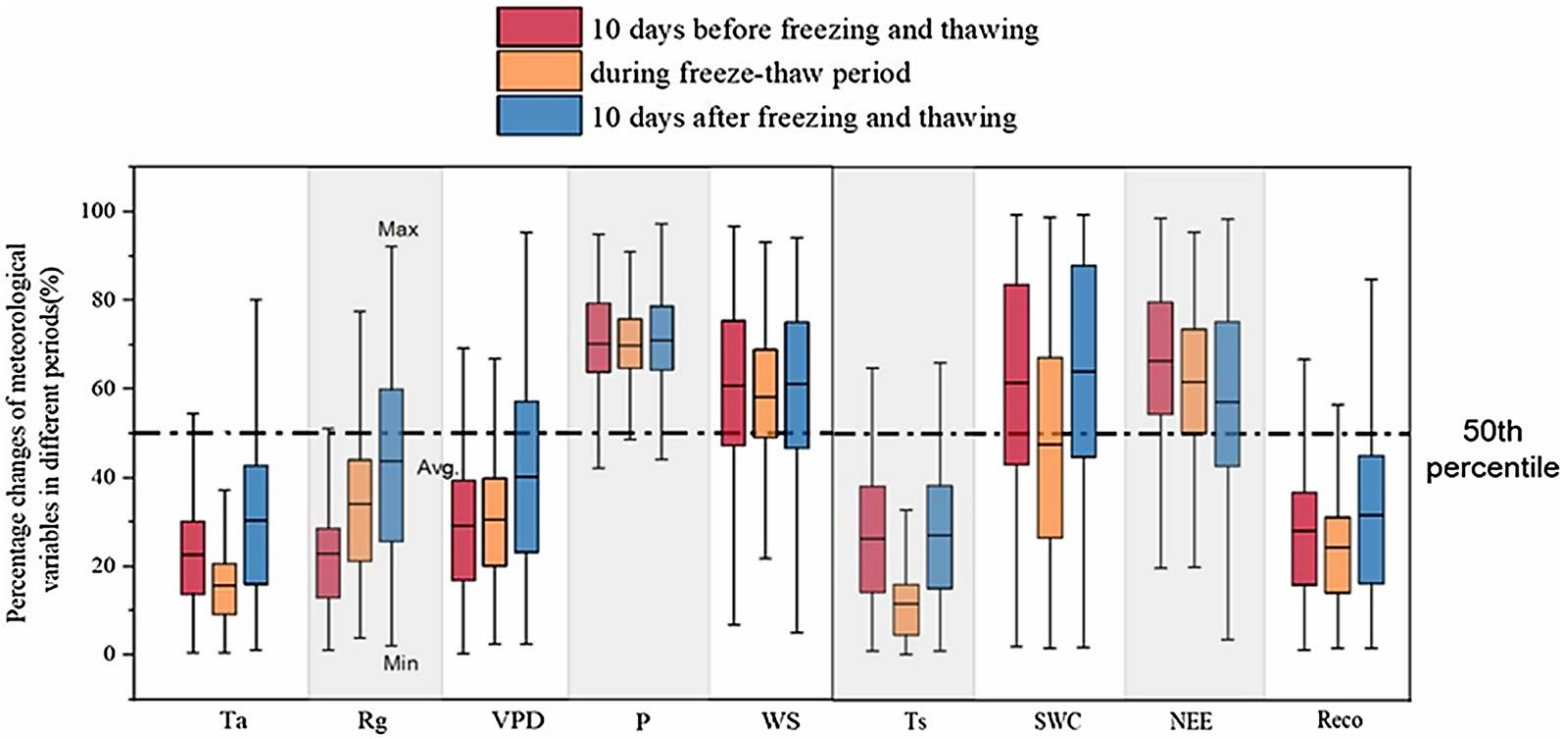
Standardized percentiles of temperature (Ta), incident shortwave radiation (Rg), vapor pressure deficit (VPD), precipitation (P), wind speed (WS), soil temperature (Ts), soil water content (SWC, %), net ecosystem carbon exchange (NEE), and ecosystem respiration (RE) during the 10 days prior to freeze–thaw events, during the freeze–thaw events, and the 10 days after freeze–thaw events.

Based on the definition of before and after freeze–thaw events in Section 2.1, we analyzed the ecological changes at different stages, including temperature, precipitation, VPD, shortwave radiation, wind speed, soil temperature, soil moisture, and standardized outliers of NEE and RE percentiles (the percentiles of the indicators at this site during the monitoring period) (Figure 3). The average percentile of ecosystem NEE in the first, middle, and last three stages of freeze–thaw events is 66.57%, 61.77%, and 57.21%, respectively. Prior to the freeze–thaw event, ecosystem respiration accounted for 28.07% of the reference level; during the event this proportion declined to 24.38%, whereas it subsequently rose to 31.75% after the freeze–thaw disturbance had ceased.

The most significant decreases during the FTC were in soil temperature and soil moisture compared to before and after the FTC. The mean percentile for soil temperature was 11.71 percentile during the freeze–thaw period, while the mean percentile before and after the FTC period was 26.33 percentile and 27.13 percentile, respectively. The mean percentile for soil moisture was 47.75 percentile during the FTC period, while the mean percentile before and after the freeze–thaw was 61.65 percentile and 64.19 percentile, respectively. At the same time, temperature, precipitation, and wind speed showed the lowest mean percentile during the freeze–thaw period.

### 3.2 Characteristics and Trends of Freeze–Thaw Events

**FIGURE 4:**
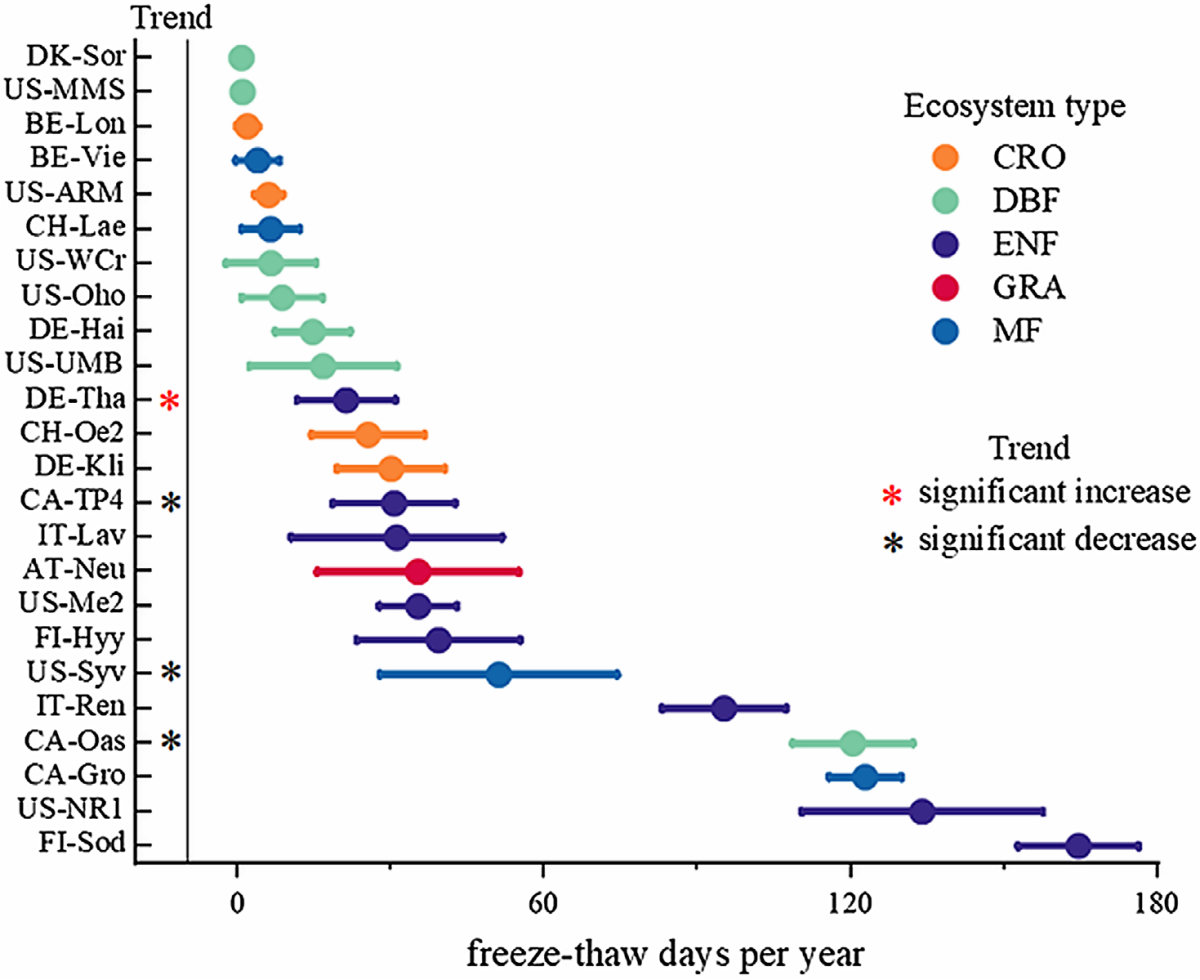
The annual average days of and trends of freeze–thaw events at different monitoring stations were classified according to different vegetation types. Only significant temporal trends (*p* < 0.05) are shown (asterisk).

Based on the average days of freeze–thaw per year at each site (Figure 4), FI‐Sod is the site with the highest average days of freeze–thaw per year, up to 164.61 days, and DK‐Sor is the site with the lowest average days of freeze–thaw per year, about 0.71 days. In terms of vegetation type, DBF and CRO have short mean annual freeze–thaw durations and ENF has the highest mean freeze–thaw duration. Among the 24 sites, the DE‐Tha site showed a significant upward trend in freeze–thaw duration; three sites, CA‐TP4, US‐Syv, and CA‐Oas, showed a significant downward trend in freeze–thaw duration, while the remaining sites showed no significant trend change.

### 3.3 Resistance of NEE and RE to Freeze–Thaw Events and Machine Learning Interpretation

The XGboost model was used to estimate ecosystem NEE and RE resistance to freeze–thaw events. The results showed that the XGboost model had a better range of variance explained for RE resistance (*R* ^2^ = 0.60, slope = 0.74, MAE = 0.072), and the results for NEE resistance were *R* ^2^ = 0.38, slope = 0.65, MAE = 0.073.

**FIGURE 5:**
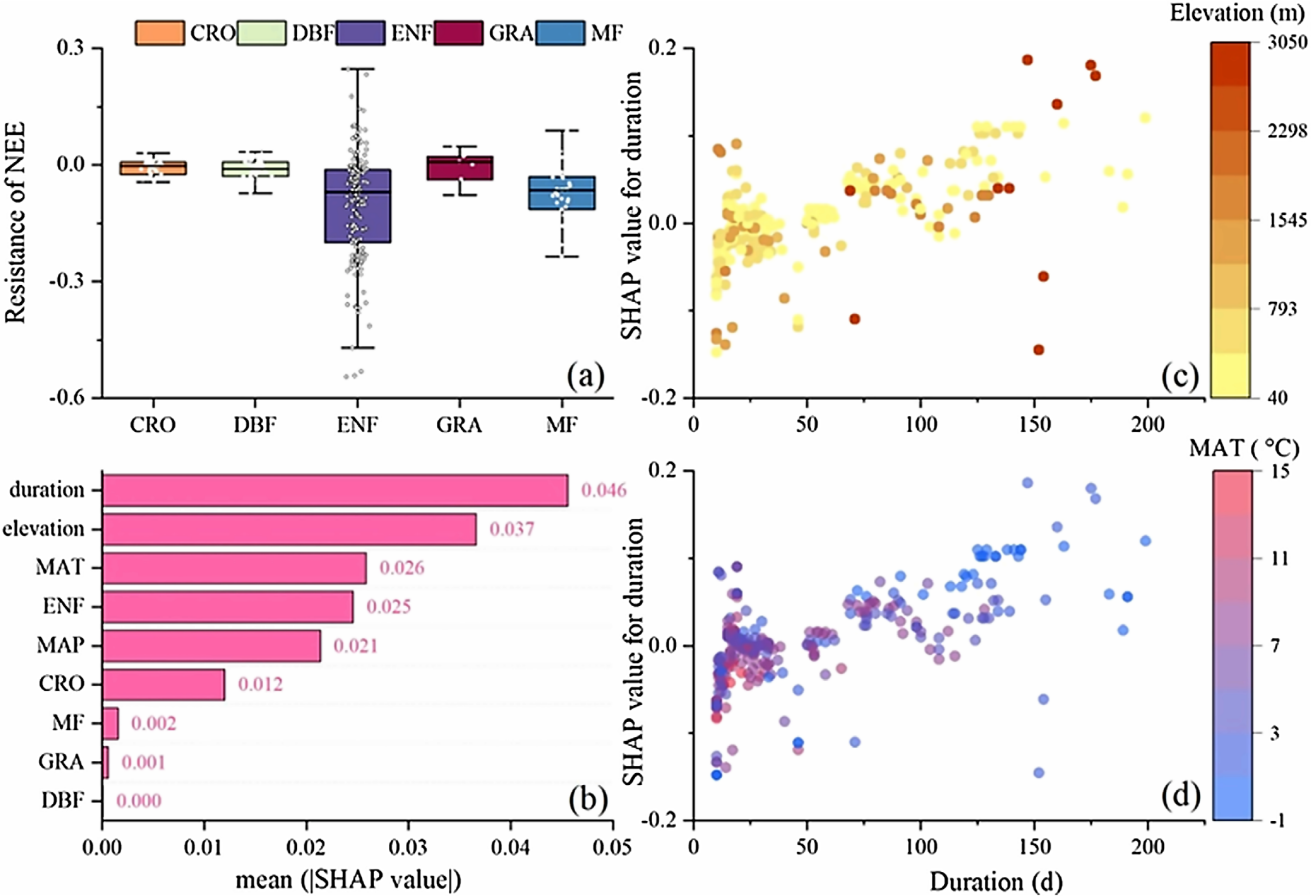
(a) Resistance of NEE to freeze–thaw events in different vegetation types. (b) SHAP values of various factors for NEE resistance (Duration, d) refers to the duration of freeze–thaw cycles, elevation refers to the altitude of the station, MAT (°C) refers to mean annual temperature, MAP (mm) refers to mean annual precipitation. (c) SHAP dependence plot of duration versus its SHAP value along a elevation gradient. (d) SHAP dependence plot of duration versus its SHAP value along a MAT gradient.

The NEE resistance of different vegetation types to freeze–thaw events is CRO (9.22 × 10^−3^), DBF (−1.43 × 10^−2^), ENF (−0.12), GRA (−1.43 × 10^−2^), MF (−6.98 × 10^−2^) (Figure 5a). The SHAP values of each factor affecting ecosystem NEE resistance to freeze–thaw events were quantified, and the duration of freeze–thaw, elevation, MAT were the three most important factors affecting NEE resistance (Figure 5b). In contrast, the interaction between the three main factors, freeze–thaw duration, elevation, and MAT, was not significant (Figure 5c).

**FIGURE 6:**
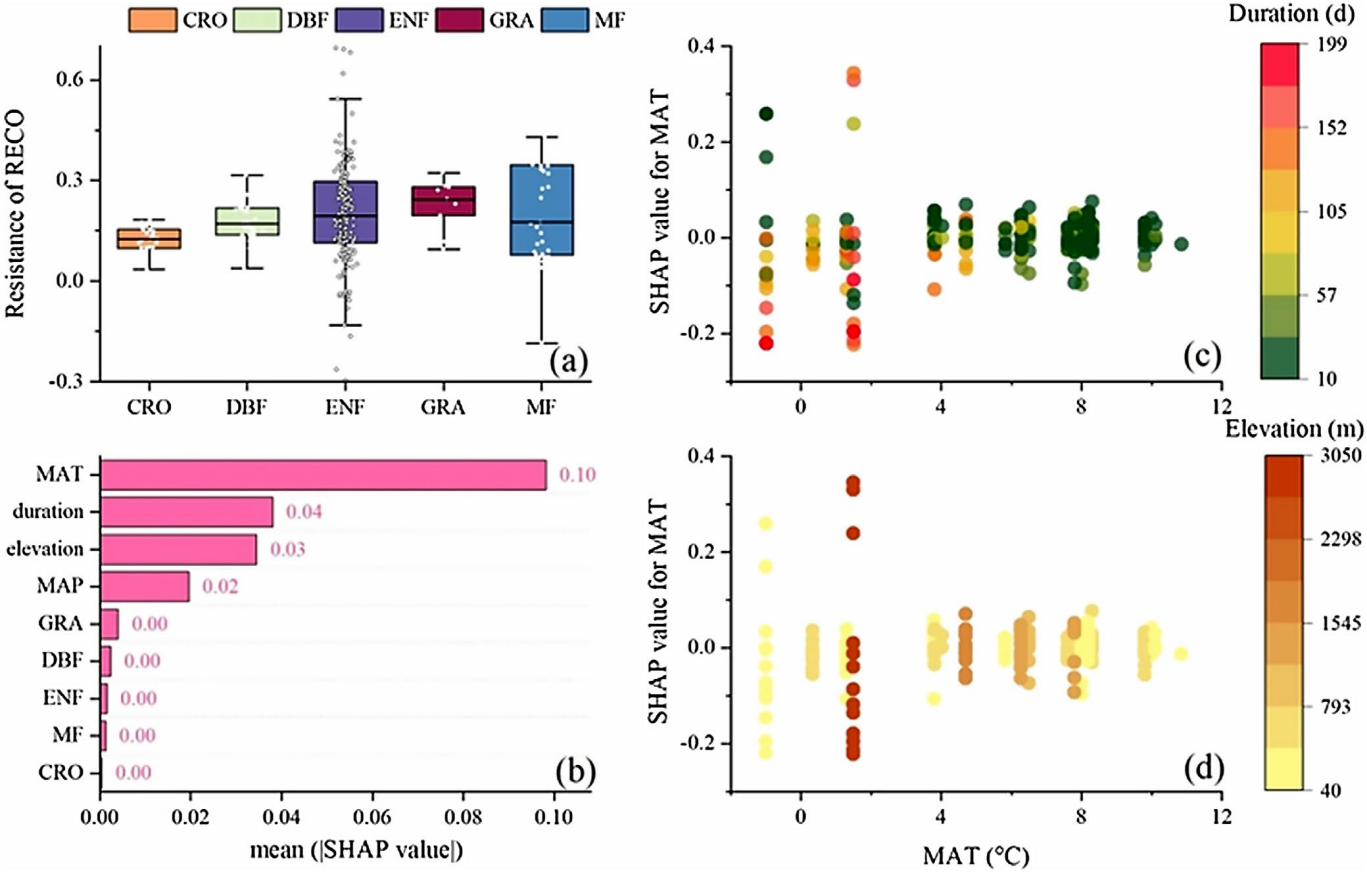
(a) Resistance of RE to freeze–thaw events in different vegetation types. (b) SHAP values of various factors for RE resistance. (c) SHAP dependence plot of MAT versus its SHAP value along a duration gradient. (d) SHAP dependence plot of MAT versus its SHAP value along an elevation gradient.

The RE resistance to freeze–thaw events of different vegetation types is CRO (0.11), DBF (0.18), ENF (0.24), GRA (0.22), MF (0.20) (Figure 6a). The SHAP values of each factor affecting RE resistance to freeze–thaw events were quantified, and MAT, the duration of freeze–thaw, and elevation were the three most important factors affecting NEE resistance (Figure 6b). There was an interaction of MAT with freeze–thaw duration, with high annual mean temperatures usually accompanied by lower freeze–thaw duration (Figure 6c). In contrast, the interaction of MAT with elevation was not significant.

## 4 Discussion

### 4.1 Impact of Freeze–Thaw Events on Ecosystems

Carbon flux variability in RE and NEE arises from a complex interplay of climate, phenology, physiology, and soil conditions (Xing et al. 2023). This study delivers a global, cross‐ecosystem assessment of how freeze–thaw cycles shape net ecosystem carbon exchange and ecosystem respiration. Soil freeze–thaw is a complex physicochemical process that couples energy balance, water transfer, and salt accumulation (Liu et al. 2021). After analysis of the changes in various environmental factors before and after freeze–thaw cycles shows that soil temperature and moisture have the most significant impact (Figure 3), which are key factors affecting microbial activity and decomposition processes in soil. The observed decrease in these parameters during FTC suggests a temporary reduction in the rate of organic matter decomposition and subsequent carbon dioxide emissions (Xiao et al. 2019). The observed suppression of ecosystem respiration during freezing aligns with earlier reports of reduced soil respiration under frozen conditions (Gao et al. 2021). The subsequent thawing period may lead to an outbreak of microbial activity and increased decomposition, resulting in an increase in ecosystem respiration intensity (Wang, Lv, et al. 2020; Rooney et al. 2022). This highlights the importance of considering the timing and intensity of FTC when assessing ecosystem carbon dynamics.

### 4.2 Drivers of NEE and RE Resistance to Freeze–Thaw Events

The results of this study indicate that RE exhibits relatively high resistance, while NEE has lower resistance.

From the perspective of respiration (RE), its resistance is mainly regulated by soil microorganisms and root physiology. During freeze–thaw cycles, the phase transition of soil liquid water and the formation of ice crystals can physically damage soil aggregates and cause partial microbial cell lysis, which typically releases readily available dissolved organic carbon to provide substrates for surviving microbial communities (Ji et al. 2022; Rooney et al. 2022). Therefore, although low temperature itself inhibits metabolic rate, the brief, pulsed substrate effectiveness may to some extent maintain or buffer the decrease in RE, thereby exhibiting a certain degree of resistance.

The key reason for the significantly lower resistance of NEE compared to RE is the extreme sensitivity and fragility of photosynthesis. The processes of snowfall and freezing that often accompany freeze–thaw events can reduce the photosynthetically active radiation received by vegetation by covering the canopy and reflecting sunlight, further limiting the progress of photosynthesis.

To further analyze the impact of freeze–thaw events on carbon exchange in ecosystems under different environmental conditions, we use interpretable machine learning to increase our understanding of freeze–thaw risks. Based on the different resistance ranges of NEE and RE, we can observe that respiration is more significantly affected by freeze–thaw events (Figures 5a and 6a). The resistance of RE to FTC, as estimated by the XGBoost model, varied among different plant functional types (PFTs). The higher resistance in croplands (CRO) and deciduous broadleaf forests (DBF) may be attributed to their ability to maintain carbon uptake and respiration processes even under the stress of FTC. This could be related to the presence of deeper root systems that access water and nutrients during thawing periods or the presence of more resilient microbial communities. In contrast, the lower resistance in evergreen needle‐leaf forests (ENF) and grasslands (GRA) suggests a higher sensitivity to FTC, which could be due to their shallower root systems or less diverse microbial communities (Grossiord et al. 2017; Shekhar et al. 2023). The duration of FTC, elevation, and annual average temperature (MAT) are the main factors affecting NEE and RE. These factors likely influence the acclimation and adaptation mechanisms of ecosystems to FTC. An extended FTC duration may provide microbial communities with a prolonged interval for thermal acclimation to fluctuating temperatures, whereas higher elevations and associated declines in mean annual air temperature are likely to constrain the overall metabolic rates of soil biota.

The differential model performance for NEE and RE resistance is a key point that warrants in‐depth discussion.

The core reason lies in the fundamental ecological difference between NEE and RE: NEE is a net flux determined by the balance between Gross Primary Production (GPP) and RE. While RE is primarily driven by soil microbial and root activity (which are strongly influenced by the environmental factors in our model, like soil temperature and moisture), NEE is additionally and significantly influenced by photosynthetic processes (GPP). During FTC, GPP can be severely suppressed by factors not fully captured in our model, such as canopy physiology, frost damage, phenological status, and light availability.

Therefore, the lower explanatory power of the NEE model (*R* ^2^ = 0.38) is not a mere methodological shortcoming but rather a reflection of this greater physiological complexity. It underscores that the net carbon exchange is shaped by the asymmetric responses of both carbon uptake (GPP) and release (RE) to FTC disturbances.

### 4.3 Methodological Considerations and Future Research

**FIGURE 7:**
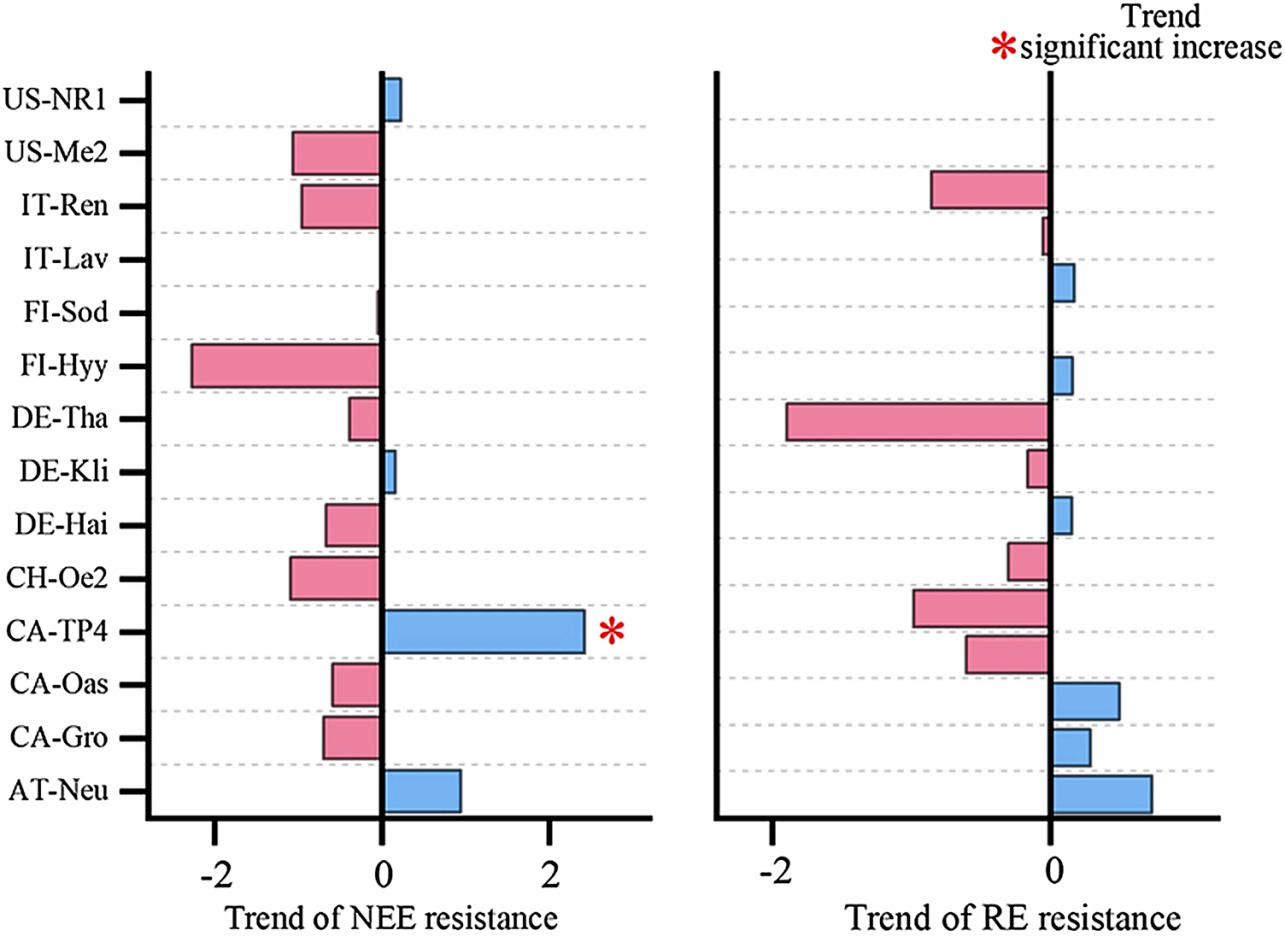
Trends in NEE, RE resistance to freeze–thaw events at each site. Only significant temporal trends (*p* < 0.05) are shown (asterisk). At several sites, the limited number of recorded freeze–thaw events precludes robust trend analysis.

According to the statistics of all stations where freeze–thaw events have occurred, the average days of freeze–thaw per year ranges from 0.71 to 164 days. However, due to the limitation of the monitoring time (10–18 years), most sites did not show significant trends in freeze–thaw days and resistance changes (Figures 3 and 7). Trend analysis only showed statistically significant trends at a limited number of sites, which may be related to small sample sizes, short time coverage, or high interannual variability. Further monitoring is needed for analysis in the future.

The variability in the annual days of FTC among FLUXNET stations underscores the regional differences in the response to climate change. The upward trend in FTC duration at the DE‐Tha site may indicate a longer period of soil thawing, which could lead to increased microbial activity and carbon emissions. Conversely, the downward trends at CA‐TP4, US‐Syv, and CA‐Oas sites suggest a potential shift towards more stable soil conditions, possibly due to changes in vegetation cover or microclimate regulation. These contrasting trends emphasize the need for a nuanced understanding of FTC impacts across different ecosystems and the development of region‐specific models to predict future carbon fluxes.

While the XGBoost model provided robust estimates of NEE and RE resistance, the variation in model performance between NEE and RE highlights the complexity of ecosystem responses to FTC. Future research should focus on improving the understanding of these responses by incorporating additional environmental variables such as soil pH, nutrient availability, and vegetation diversity. Long‐term observational data, combined with advanced modeling techniques, will be crucial for enhancing the predictive capacity of such models and for developing more accurate projections of ecosystem carbon dynamics under changing climate conditions.

## 5 Conclusion

This study comprehensively analyzed the effects of FTC on NEE and RE in different ecosystems based on flux monitoring data from the FLUXNET database. The average percentile of ecosystem NEE in the first, middle, and last three stages of freeze–thaw events is 66.57%, 61.77%, and 57.21%, respectively. Prior to the freeze–thaw event, ecosystem respiration accounted for 28.07% of the reference level, during the event this proportion declined to 24.38%, whereas it subsequently rose to 31.75% after the freeze–thaw disturbance. Resistance is defined as the capacity of an ecosystem to maintain stability during a freeze–thaw cycle. It is quantified as the ratio of the change in ecosystem carbon exchange before and during the freeze–thaw event to the corresponding change in soil temperature. The NEE resistance of different vegetation types to freeze–thaw events is croplands (9.22 × 10^−3^), deciduous broadleaf forests (−1.43 × 10^−2^), evergreen needle‐leaf forests (−0.12), grasslands (−1.43 × 10^−2^), mixed forests (−6.98 × 10^−2^). The RE resistance to freeze–thaw events of different vegetation types is croplands (0.11), deciduous broadleaf forests (0.18), evergreen needle‐leaf forests (0.24), grasslands (0.22), mixed forests (0.20). Soil temperature, duration of freeze–thaw events, and elevation are the main factors affecting the resistance of NEE and RE. According to the statistical data of all sites, the average days of in which freeze–thaw events occur ranges from 0.71 to 164 days. However, due to limited monitoring time (10–18 years), most sites do not show significant trends in freeze–thaw days and resistance changes. Our research provides valuable insights into the impact of FTC on ecosystem carbon exchange and the resistance of different ecosystems to these disturbances. These results are of great significance for parameterizing the freeze–thaw resistance coefficient of specific plant types to estimate winter CO_2_ flux and improve the prediction of carbon release in permafrost regions. In terms of ecological protection in the future, priority should be given to deep rooted deciduous tree species or winter tolerant varieties to maintain soil carbon absorption in warmer and more unstable winters, while protecting high‐altitude mixed forests can help maintain the buffering capacity of natural freeze–thaw cycles and limit carbon loss under sustained climate change.

## Author Contributions

**Qingfeng Xu:** conceptualization (equal), methodology (equal), software (equal), visualization (equal), writing – original draft (equal), writing – review and editing (equal). **Kai Guo:** funding acquisition (equal), supervision (equal). **Jintao Zhao:** conceptualization (equal), data curation (equal), visualization (equal). **Chunjing Zhao:** data curation (equal), supervision (equal). **Yinan Wang:** formal analysis (equal), supervision (equal). **Fengying Jin:** methodology (equal), writing – review and editing (equal).

## Funding

This work was supported by Natural Science Foundation of Henan Province (252300420863), National Natural Science Foundation of China (U2443214), and Central Nonprofit Research Institutions Basic Scientific Research Special Fund form Yellow River Institute of Hydraulic Research (HKY‐YF‐2024‐03, HKY‐JBYW‐2023‐08, HKF202404).

## Conflicts of Interest

The authors declare no conflicts of interest.

## Data Availability

Data used for model fitting are available at can be obtained from the website (https://figshare.com/articles/dataset/fluxdata_zip/29817584).
